# The proper timing of second-stage revision in treating periprosthetic knee infection: reliable indicators and risk factors

**DOI:** 10.1186/s13018-018-0885-z

**Published:** 2018-08-29

**Authors:** Jun Fu, Ming Ni, Heng Li, Xiang Li, Wei Chai, Yonggang Zhou, Libo Hao, Jiying Chen

**Affiliations:** 1 Department of Orthopaedics, Chinese People’s Liberation Army General Hospital (301 Hospital), Beijing, 100853 China; 2 Department of Orthopaedics, Beijing Jishuitan Hospital, Beijing, China

**Keywords:** Periprosthetic joint infection, Total knee revision, Two-stage revision, Serum CRP, ESR, Intraoperative frozen section

## Abstract

**Background:**

Two-stage revision is the standard procedure for periprosthetic knee infection. But when to perform the second-stage is still under debate. We attempt to search the reliable indicators, risk factors, and proper timing for the second-stage revision.

**Methods:**

We reviewed and followed 81 infected total knee arthroplasty patients who underwent two-stage revision from January 2010 to January 2014. Our cohort included 56 males and 25 females, all patients were confirmed as PJI with the same phenotypic cultures. The average age was 64.8 ± 8.21 (range 36–78) months. The mean follow-up time was 46.5 ± 17.6 (range 12–72) months after the second-stage surgeries. The diagnostic parameters including serum C-reaction protein, erythrocyte sedimentation rate, and intraoperative frozen section at the time of re-implantation were analyzed. The spacer detention time and antibiotic treatment time were compared.

**Results:**

Ten of them went through failed first- or second-stage surgeries. The overall success rate was 87.7%. The intraoperative frozen section is a good indicator at the time of re-implantation; the sensitivity and specificity is 90 and 83.1%. Serum CRP and ESR showed poor diagnostic value at time of re-implantation. Atypical pathogen infection, positive FS, and previous sinus were high-risk factors for failure of two-stage revision. Spacer detention time between 12 and 16 weeks had higher success rate than over 16 weeks.

**Conclusion:**

The proper timing of re-implantation should be combined with disappearance of clinical symptoms and negative intraoperative FS with spacer detention time at 12 to 16 weeks.

## Background

Periprosthetic joint infection (PJI) is the most devastated complication after total knee arthroplasties. The treatment option of PJI involves one- and two-stage revision. The standard procedure of one-stage revision includes the removal of infected prosthesis, thorough debridement and exchange of a new prosthesis. The two-stage revision requires antibiotic-loaded cement spacer implantation in the first stage and intravenous antibiotic treatment before the second stage. During the last two decades, both of the two protocols were reported with satisfied infection eradication rates. However, which treatment is more appropriate for patients depends on their clinical status, culture results, general health conditions, and even financial situations.

All the therapeutic efforts made before would be all in vain with the catastrophic recurrence of PJI. Sometimes failure is hard to avoid. The success rates of the two-stage revision vary from 4 to 41% [1]. This discrepancy of the reinfection rates could be attributed to inconsistent study factors including patient demographic characteristics, pathogenic characteristics, and different treating protocols. Moreover, no reliable reference standard for re-implantation is another cause. Therefore, how to rule out PJI at the time of second-stage revision is the key to a successful two-stage revision.

So far, there is no authentic method to evaluate the control of PJI at the time of second-stage surgery. And there is also no clinical trial aiming at the best timing of second stage. Usually, surgeons had to make decisions basing on clinical symptoms combined with laboratory parameters. While in some cases, they can only rely on their own experiences. But, it is reported serologic markers such as C-reaction protein (CRP) and erythrocyte sedimentation (ESR) are not reliable markers at the time of second-stage surgery [2, 3]. Although these laboratory indicators do decrease after intravenous antibiotic treatment, it is still difficult to rule out persist PJI. Elie reported both CRP and ESR poorly predicted the persistent infection and the cut-off values were difficult to obtain [3]. Some authors suggested using the Musculoskeletal Infection Society (MSIS) criteria to assess infection at the time of second stage. It has been reported that both MSIS criteria and frozen sections have high specificity for ruling in failure [2]. As intraoperative synovial or tissue cultures are not available immediately, it could not provide convincing support at the time of re-implantation [4–6]. These limitations make intraoperative frozen section a better reference in judging infection at the time of re-implantation [7, 8].

Failed two-stage revisions discourage both patients and doctors. Under such circumstance, we desperately need to find out the reliable indicators and risk factors of these failed cases. We reviewed our recent two-stage total knee revision patients with positive culture results, and try to answer the following questions: (1) are intraoperative frozen sections, serum CRP, and ESR reliable references for the second-stage revision? (2) What are the risk factors for the failure of two-stage revision? (3) What is the proper timing for the second-stage revision?

## Methods

Eighty-one periprosthetic knee infection patients from January 2010 to January 2014 with positive culture results were followed in this retrospective study. All patients received two-stage revision with articulated antibiotic-loaded cement spacer implantation. We have excluded patients with negative culture results, as they were hard to diagnose as PJI even with the MSIS criteria. Our cohort included 56 male and 25 female, the average age was 64.8 ± 8.21 (range 36–78) years. The mean follow-up time was 46.5 ± 17.6 (range 12–72) months after the second-stage surgeries. This study was approved by the institutional review board of our hospital, and all participants were informed and content.

All these patients showed suspicious symptoms of infection including periprosthetic fever, swelling or pain when they came to our clinic. Their medical histories were collected and laboratory tests including serum CRP and ESR were ran to help diagnose PJI. The threshold of CRP and ESR was 10 mg/L and 30 mm/h according to MSIS criteria, respectively. Synovial fluid aspirations and cultures were performed. The MSIS criteria were adopted to diagnose PJI. All these 81 patients were confirmed as PJI with the same phenotypic cultures. During the first stage, the former prosthesis was removed and a radical debridement was performed. At least three periprosthetic tissues were sent as culture samples. Another three periprosthetic tissues were collected as frozen section samples. Then, we mixed sensitive antibiotic into bone cement and made handcrafted spacers. The spacers were articulated in order to maintain soft tissue balancing. After the spacer was implanted, sensitive intravenous antibiotic treatment initiated. This treatment period lasted for at least 6 weeks. If the culture results suggested pathogens, such as fungal or mycobacterium tuberculosis, were hard to eradicate, the antibiotic treatment would extend. Patients were followed by phones after the spacer implantations until they finished the intravenous antibiotic treatment. The infection was considered as being eradicated with no clinical signs and symptoms, then they came back to our hospital for re-evaluation.

Before the second stage, serum CRP and ESR were tested again to reassess the control of infection and inflammation in the tissues. During the second-stage surgeries, at least three periprosthetic frozen sections and three tissue culture samples were collected after the removal of cement spacers. We employed the Feldman criterion for frozen section, which was interpreted as more than five neutrophils per high-power field (× 400) in at least five separate microscopic fields [9]. The following step depended on the overall consideration by surgeons, either implantation of a new prosthesis or another cement spacer.

These following situations were regarded as failure: (1) repeated spacer implantation; (2) recurrent infection with the same pathogen after new prosthesis implantations.

### Statistical analysis

All data were entered into database and analyzed by SPSS software (Version 19.0. NY, USA). Quantitative data including age, height, weight, and BMI were described with mean and standard deviation (SD). The follow-up time, spacer detention time, and survival time were reported as median and range. Categorical data were described with percentages. We used Students’ *t* test to compare the difference of age, height, weight, BMI, and spacer detention time. Chi-square test was used to identify the difference of qualitative parameters. We also calculated the sensitivity and specificity of CRP, ESR, and Frozen section at time of second-stage revision. Receiver operating characteristic (ROC) curves were drawn to elevate the diagnostic accuracy of CRP, ESR, and frozen section at the second-stage revision. The area under the ROC curves (AUC) was also calculated. Cox proportional hazards model were adopted to explore the risk factors for failed two-stage surgeries. The *P* values less than 0.05 were considered as statistically significant.

## Result

### Diagnose of PJI and detected microorganisms

The major clinical symptom of the cohort was persistent pain with impaired joint function. According to the pain grading of World Health Organization (WHO), 67 (82.72%) patients were at grade II, while the rest 14 patients (17.28%) were at grade III. Sinus tract was observed in 17 patients (21%). Pre-operative joint aspiration culture identified pathogens in 52 patients before the first stage surgeries. And the other microorganisms were detected through the intra-operative tissue cultures. The most common pathogens were Staphylococcus, and 31.5% of them were methicillin-resistant. The atypical pathogens of PJI were detected in 27 patients. There were also nine patients infected with fungus including five *Candida parapsilosis*, three *Candida albicans*, and one aspergillus. Other microorganisms included Streptococcus and *Escherichia coli* (Table 1).

**Table 1:** Microorganisms detected in 81 periprosthetic knee infection patients

	Organism detected	*N*	Percentage
Non methicillin-resistant	*Staphylococcus aureus*	12	14.8%
	*Staphylococcus epidermidis*	10	12.3%
	Coagulase-negative Staphylococcus	12	14.8%
	*Staphylococcus warneri*	3	3.7%
Methicillin-resistant Staphylococcus	MRSE	9	11.1%
	MRSA	8	9.88%
Fungus	*Candida albicans*	3	3.7%
	*Candida parapsilosis*	5	6.17%
	*Aspergillus*	1	1.23%
Other pathogen	Streptococcus	4	4.94%
	*Escherichia coli*	5	6.17%
	*Pseudomonas aeruginosa*	1	1.23%
	*Enterococcus faecalis*	2	2.47%
	*Mycobacterium tuberculosis*	2	2.47%
	NTM	2	2.47%
	*Klebsiella pneumoniae*	1	1.23%
	*Acinetobacter baumannii*	1	1.23%

*MRSA* methicillin-resistant *Staphylococcus aureus*, *MRSE* methicillin-resistant *Staphylococcus epidermidis*,

*NTM* non-tuberculosis mycobacterium

### Demographic information

There were 71 patients (22 males and 49 females) received primary total knee arthroplasties for end-stage osteoarthritis, 5 (2 males and 3 females) for traumatic arthritis, and 5 (4 males and 1 female) for rheumatoid arthritis. The average age at primary TKA was 64.8 ± 8.21 years. The average height, weight, and BMI were 1.62 ± 0.07 m, 69.5 ± 11.6 kg, and 26.31 ± 3.6 kg/m^2^, respectively. All these patients were followed after each stage surgery. The mean follow-up time after the second-stage revision was 46.5 ± 17.6 months (range 12–72 months). The mean spacer detention time was 24.02 ± 16.6 weeks (range 12–96 week). The mean antibiotic treatment time was 7.59 ± 2.54 weeks (range 4–12 weeks).

### Unexpected repeated first-stage and failed second-stage revision

The general information of patients with successful and failed two-stage revisions was compared with Students’ *t* test or chi-square test. No significant difference was found regarding age, height, weight, BMI, and pain severity. And there was also no difference of follow-up time (Table 2).

**Table 2:** Comparison between successful and failed two-stage patients

	Successful two-stage	Failed two-stage	*p* value
Number	71	10	*N*
Age (years)	65.14 ± 8.45	62.4 ± 6.08	0.326
Height(m)	1.62 ± 0.06	1.65 ± 0.09	0.147
Weight(kg)	69.18 ± 11.18	72.10 ± 14.76	0.461
BMI(kg/m^2^)	26.30 ± 3.51	26.42 ± 4.82	0.919
Pain severity			0.171
I	42	3	
II	20	4	
III	9	3	
Follow-up time (months)	54.92 ± 27.03	43.0 ± 25.30	0.191
Spacer detention time (weeks)	23.97 ± 17.28	24.4 ± 11.54	0.94
Antibiotic treatment time (weeks)	7.23 ± 2.14	10.20 ± 5.69	0.135

Seventy-one patients were successfully cured with two-stage protocol. The success rate of two-stage revision was 87.7%. During our investigation, we observed five patients with failed first-stage surgeries and five with failed second stages. Five patients with failed first-stage revisions underwent repeated cement spacer implantation. And two of them were infected with non-tuberculosis mycobacterium, and they all received three spacer implantations before the final prostheses were implanted. The other five patients had reinfection with same pathogen after replacements of new prostheses. There were three patients that had sinus tract recurrence after second-stage revisions, two of them were infected with methicillin-resistant Staphylococcus, the other one was infected with *Candida albicans* (Table 3).

**Table 3:** Information of failed second-stage revisions

Number	Gender	Age	BMI	Diagnosis	Pathogen	Spacer retention period (weeks)	Failed details	Ending
4	Male	67	22.4	OA	MRSE	24	Sinus recurrence 9 months after spacer implanted	Three spacer implantations, one I&D, infection controlled.
16	Male	75	17.8	OA	MRSA	12	Sinus recurrence 6 months after prosthesis implanted	Two I&D and insert exchange, fusion
18	Female	65	27.7	OA	*Enterococcus faecalis*	12	Reinfection 39 months after new prosthesis implanted	Another one-stage revision, infection controlled
23	Female	56	26.6	OA	*Group G Streptococcus*	24	Infection persist 2 months after spacer implanted	Two spacer implantation, Two I&D and insert exchange, infection controlled
29	Female	63	23.9	RA	*Candida albicans*	48	Infection persist 16 months after spacer implanted	Two spacer implantation, infection controlled
30	Female	59	28.9	OA	MRSE	20	Reinfection 24 months after prosthesis implanted	Another one-stage revision, infection controlled
32	Male	64	22.5	OA	NTM	24	Infection persist 9 months after spacer implanted	Three spacer implantations, infection controlled.
35	Female	58	31.3	OA	*Candida albicans*	40	Sinus recurrence 6 months after prosthesis implanted	One I&D and insert exchange, infection controlled
37	Male	63	34.3	OA	NTM	24	Infection persist 10 months after spacer implanted	Three time spacer implantations, infection controlled
57	Male	54	28.7	OA	*Candida parapsilosis*	16	Reinfection 4 months after prosthesis implanted	One I&D and insert exchange, infection controlled

*MRSA* methicillin-resistant *Staphylococcus aureus*, *MRSE* methicillin-resistant *Staphylococcus epidermidis*, *NTM* non-tuberculosis mycobacterium, *I&D* irrigation and debridement

### Spacer detention time

In our study, we discovered 16 week of spacer detention time is a valuable point for re-implantation. Forty patients received new prosthesis implantation with the spacer detention time between 12 and 16 weeks, and only three of them failed. The other 41 patients had new prostheses implanted with more than 16 weeks, and 7 of them endured failed two-stage revision. The difference was statistically significant (7.5 vs 17.1%, *p* = 0.001).

### Reliable indicators at re-implantation

**Fig. 1:**
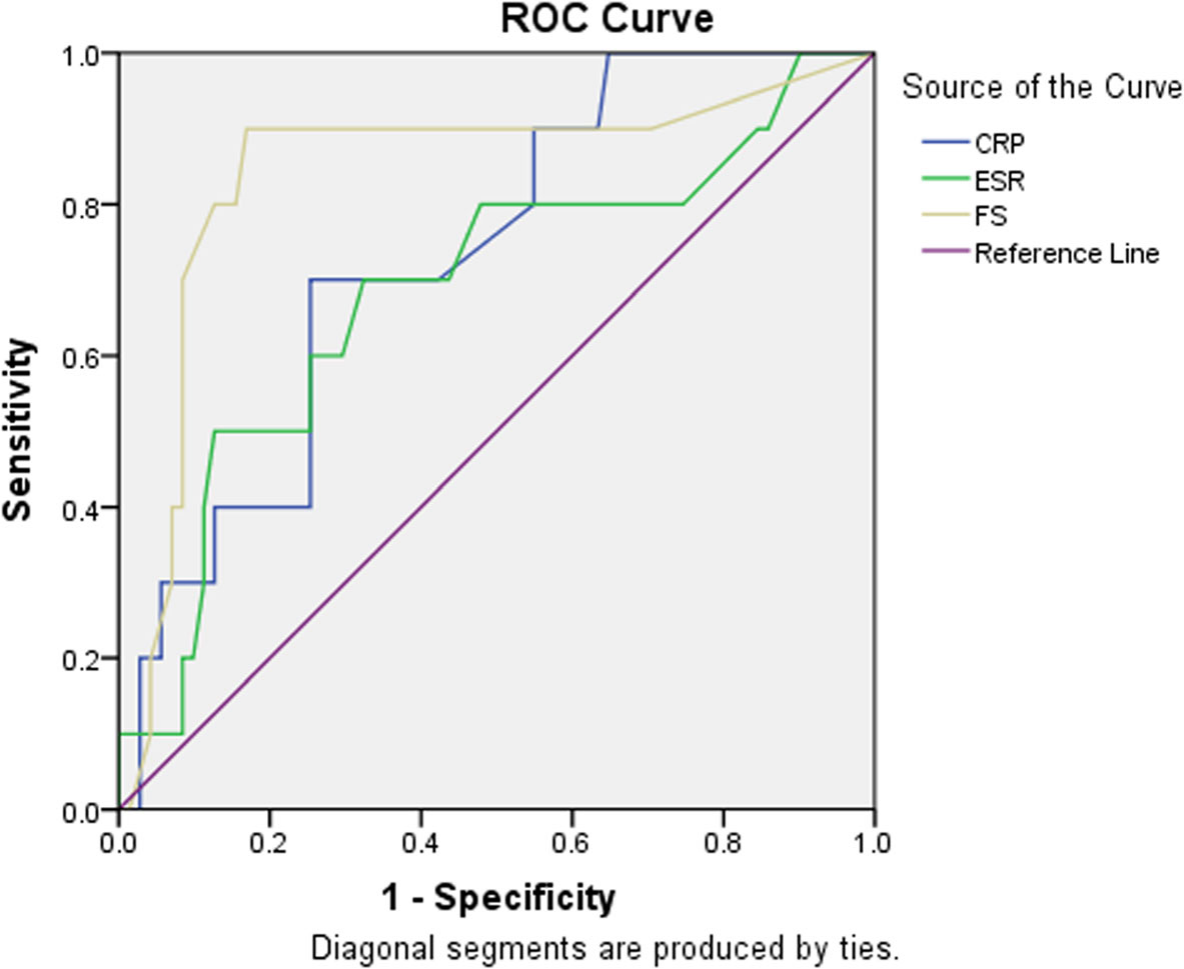
ROC curve of intraoperative frozen section, serum CRP, and ES

We evaluated the diagnostic value of serum CRP, ESR, and intraoperative frozen section at the time of re-implantation. The AUCs proved both intraoperative frozen section was the most useful diagnostic indicator (FS: AUC = 0.84, *p* < 0.05, Fig. 1). While serum ESR was not available as diagnostic parameters at the time of re-implantation (AUC = 0.69, *p* = 0.055).

At the time of re-implantation, intraoperative frozen section showed outstanding diagnostic capacity of both ruling out and ruling in infection with the sensitivity was 90% (95% confidence interval, 54.1–99.5%) and specificity was 83.1% (95% confidence interval, 71.9–90.6%). The overall accuracy of FS was 84%. Both serum CRP and ESR were useful in ruling in infection where the specificity was 88.7% (95% confidence interval, 78.5–94.7%) and 84.5%(95% confidence interval 73.5–91.6%); while they had poor utility in ruling out infection with sensitivity that was only 40% (95% confidence interval, 13.7–72.6%) and 50% (95% confidence interval, 20.1–80.0%) (Table 4). The Youden Index of frozen section was 0.73. This result indicated serum ESR and CRP were not good diagnostic indicators at the time of re-implantation with the Youden Index were 0.29 and 0.35, respectively.

**Table 4:** Diagnostic evaluation of CRP, ESR and Frozen section at re-implantations

CRP	40% (13.7–72.6%)	88.7% (78.5–94.7%)	33.3% (11.3–64.6%)	91.3% (81.3–96.4%)	82.70%	0.732	0.29
ESR	50% (20.1–80.0%)	84.5% (73.5–91.6%)	31.3% (12.1–58.5%)	92.3% (82.2–97.1%)	80.20%	0.688	0.35
FS	90% (54.1–99.5%)	83.1% (71.9–90.6%)	42.3% (22.6–65.6%)	98.3% (89.9–99.9%)	84.00%	0.843	0.73

*PPV* positive predictive value, *NPV* negative predictive value, *AUC* area under the curve

### Risk factors for failed two-stage TKA revision

Our Cox proportional hazards model revealed that positive frozen section, atypical pathogen infection, and previous sinus tract were the risk factors for failed two-stage revisions (Table 5). If the patient had a sinus tract communicating to the joint, the failure rate for two-stage revision would increase 7.94 times (hazard rates[HR],7.94[1.701–37.093], *p* = 0.008). At the time of re-implantation, if the frozen section shows more than five neutrophils per high-power field (× 400 magnification), the failure rate would increase 4.2 times (hazard rates [HR], 4.215 [1.047–16.959], *p* = 0.043). Patients infected atypical pathogen, the failure rate would increase 8.8 times (hazard rates [HR], 8.833[1.165–66.949], *p* = 0.035). However, antibiotic treatment less than 6 weeks, positive serum CRP and ESR were not risk factors for failure.

**Table 5:** Risk factors for failed two-stage revision

Variables in the equation								
	B	SE	Wald	df	Sig.	Exp(B)	95.0% CI for Exp(B)	
							Lower	Upper
Sinus	2.072	0.786	6.944	1	0.008	7.942	1.701	37.093
Antibiotic treatment less than 6 weeks	− 0.596	0.978	0.372	1	0.542	0.551	0.081	3.746
Positive CRP	0.313	0.835	0.141	1	0.708	1.368	0.266	7.029
Positive ESR	1.276	0.787	2.629	1	0.105	3.582	0.766	16.753
Positive FS	1.439	0.710	4.102	1	0.043	4.215	1.047	16.959
Atypical pathogen infection	2.178	1.033	4.444	1	0.035	8.833	1.165	66.949

## Discussion

For a long period before the MSIS criteria, the diagnosis of PJI was quite challenging. Over the past several years, lots of researchers have proved the clinical value of MSIS criteria in diagnosing primary PJI [10]. Despite of many efforts on diagnosing of primary PJI, researchers seldom focused on the re-evaluation methods for the proper timing of re-implantation. As so far, there existed no consensus on this issue. However, the timing of re-implantation deserves more attention.

In our study, we have excluded suspicious PJI patients without pathogenic results, as these patients were hard to confirm as infection even with MSIS criteria [6, 11]. All selected 81 patients had positive culture results, PJI diagnoses were undisputed. The major microorganism detected was Staphylococcus, and 20% of them were methicillin-resistant Staphylococcus. Our cohort also involved some rare pathogens, such as NTM and *Candida albicans*. At mean 46.5 months follow-up, the overall successful rate of two-stage revision in this study was 87.7%, which was consistent with previous published researches in this field [1, 12]. We observed 10 patients failed in the two-stage revision, among which there included three Candida and two NTM-infected patients. Our COX model indicated that atypical-infected TKA patients were 8.8 times more likely to fail a two-stage revision. All the 10 patients received multiple unexpected surgeries, which reminded us the high risk of failure when the pathogen was rare and needed longtime and combined antibiotic treatment.

To our acknowledgment, most surgeons judge the timing of re-implantation by clinical symptoms and certain laboratorial indicators. Our surgeons also followed this principle. But surgeons had to re-implant prostheses with risks under some circumstances, for example, patients were unable to afford or bear another spacer exchange. In our study, six patients underwent re-implantation with persistently elevated serum CRP and ESR, and eventually half of them failed.

The length of antibiotic treatment is very important for treating PJI. Bernard conducted a prospective non-randomized study, and he found that 6 weeks of antibiotic treatment was sufficient for PJI [13]. Current studies suggested antibiotic treatment duration time should be held between 6 and 12 weeks [14]. In this study, the mean antibiotic treatment time between two stages was 7.11 ± 2.07 weeks, and no difference was found between successful and failed patients. We found antibiotic treatment less than 6 weeks was not a relative risk factor for failed two-stage revision in our study (*p* = 0.542). We believe 6 weeks of antibiotic treatment was enough for treating infected TKA patients.

According to Ines, the optimal timing for re-implantation was 4 to 11 weeks after the first stage. In our study, the mean spacer detention time was 24 weeks for successful two-stage revision and 21 weeks for the 10 failed [15]. However, we discovered patients with spacer detention time between 12 and 16 weeks had lower failure rate. The least detention time in our study was 12 weeks, which made it impossible to analyze the actual effect of any shorter detention time. Ines’s study included culture negative patients, and the mean follow-up time was 20.5 months. We believe this discrepancy may need further investigation and follow-up.

Improvement of symptoms alone was not enough to determine the infectious status. Most surgeons made decisions on the decrement of inflammatory indicators. As joint aspiration samples may be difficult to obtain for some patients, especially for patients with sinus. Such situation made synovial white cell count and leukocyte strip difficult to complete for certain patient. Thus, serum CRP, ESR, and intraoperative frozen sections turned out to be more convenient indicators for the verdict of infection. At the time of re-implantation, our study showed that serum CRP and ESR had low sensitivity in ruling out infection. These two indicators showed poor diagnostic value in predicting failure of two-stage revision. It is speculated that persistent infection may produce limited efforts in laboratory parameters, and the cut-off values should be reevaluated.

In this study, the sensitivity and specificity of intraoperative FS is 90% and 83.1%, and COX model revealed that positive frozen section is a high risk factors (HR 4.22, *p* = 0.043), which makes us believe FS is an excellent parameter at the time of re-implantation. According to former meta-analysis, intraoperative FS was reported as a good indicator for predicting failure in culture-positive PJI patients [16]. Again, our result confirmed this conclusion through exclusion of culture negative patients. The sinus tract was found as a high risk factor for failure (HR 7.94, *p* = 0.008). The sinus is usually generated by high virulence microorganisms, such as *Staphylococcus aureus* and Streptococcus. It means we should be more careful when treating PJI patients with sinus. The most common microorganism detected in PJI patients was Staphylococcus which usually account for nearly 40–50% [14, 17]. Our study also presented the similar culture results with Staphylococcus infection rate 66.7%. But we also detected 33.3% patients had atypical pathogen infection. Statistical analysis showed atypical pathogen PJI was a high risk factor for failed two-stage revision. Atypical pathogens of PJI patients are sometimes very difficult to incubate. Candida could grow on most medium, while NTM and fungal are high selective of growth environment, which requires special medium and prolonged culture time. Current literatures supported two-stage revision as the surgical management for these part patients [17–20].

Our study had some limitations. Firstly, this retrospective study only evaluated diagnostic efficiency of serum CRP, ESR, and FS. The negative culture result was excluded for a more authentic PJI cohort, whereas may lose some infected patients. More factors should be included to estimate inflammation of the patients. Secondly, the follow-up time is 46 months, which is relatively short for assessment of late reinfection and need further follow-up. Thirdly, the diet during the follow-up investigations is not taken into account. The specific diets which could prevent infections may be helpful for the surgical patients. Another deficiency is the low sample size; we only observed 10 failed two-stage revision patients, and it requires large sample to verify our results. And more prospective studies on timing of the re-implantation should be conducted.

## Conclusion

The intraoperative frozen section is a good indicator at the time of re-implantation; the sensitivity and specificity is 90 and 83.1%. Serum CRP and ESR showed poor diagnostic value at time of re-implantation. Atypical pathogen infection, positive FS, and previous sinus were high risk factors for failure of two-stage revision. The antibiotic treatment less than 6 weeks would not increase the failure rate. Spacer detention time between 12 and 16 weeks had higher success rate than over 16 weeks. The proper timing of re-implantation should be combined with disappearance of clinical symptoms and negative intraoperative FS with spacer detention time at 12 to 16 weeks.

## Abbreviations

AUC: Area under the ROC curves
CRP: C-reaction protein
ESR: Erythrocyte sedimentation
MSIS: Musculoskeletal Infection Society
PJI: Periprosthetic joint infection
ROC: Receiver operating characteristic
SD: Standard deviation
